# Root-zone fertilization improves crop yields and minimizes nitrogen loss in summer maize in China

**DOI:** 10.1038/s41598-018-33591-9

**Published:** 2018-10-11

**Authors:** Chaoqiang Jiang, Dianjun Lu, Chaolong Zu, Jianmin Zhou, Huoyan Wang

**Affiliations:** 1 State Key Laboratory of Soil and Sustainable Agriculture, Institute of Soil Science, Chinese Academy of Sciences, Nanjing, 210008 P. R. China; 2 Tobacco Research Institute/Maize Research Institute, Anhui Academy of Agricultural Sciences, Hefei, 230031 P. R. China

## Abstract

It is urgently to minimize nitrogen (N) loss while simultaneously ensuring high yield for maize in China. A two-year field experiment was conducted to determine the effects of root-zone fertilization (RZF) and split-surface broadcasting (SSB) on grain yield, N use efficiency (NUE), and urea-^15^N fate under different N rates (135, 180 and 225 kg ha^−1^). Results showed that RZF increased grain yield by 11.5%, and the N derived from fertilizer (Ndff%) by 13.1–19.6%, compared with SSB. The percentage of residual ^15^N in the 0–80 cm soil was 37.2–47.4% after harvest; most ^15^N (64.4–67.4%) was retained in the top 20 cm. RZF significantly increased the N apparent recovery efficiency (NARE) and ^15^N recovery in maize by 14.3–37.8% and 21.9–30.0%, respectively; while decreased N losses by 11.2–24.2%, compared with SSB. The RZF of urea can be considered a slow-release fertilizer, which better matches maize N demand and effectively reduces N losses. Overall, RZF achieved yields as high as the SSB, but with a 20–25% reduction in N application. These results help improve our understanding of N fate in the maize cropping system, and may help guide recommendations for N management in southeastern China.

## Introduction

Nitrogen (N) fertilizer plays an important role in crop yields and is therefore relevant to food security^1,2^. The global demand for N fertilizer in agriculture has consistently increased^3^ and is estimated to increase by 100–110% between 2005 and 2050 to meet the needs of crop production^4,5^. Excessive use and inappropriate application methods of N fertilizer have led to high N losses, such as ammonia volatilization, leaching (i.e., removal by drainage water) and denitrification (i.e., transformations into gaseous forms)^1,3,6^, resulting in low N use efficiency (NUE) and various environmental problems^7,8^. Therefore, managing N fertilizer for efficient application, specifically to increase crop yields while reducing N losses, is an important scientific issue tied to both food security and environmental problems^1,9^.

Over-application of nitrogen fertilizer is common in China when farmers attempt to increase yields^1,10^. For agricultural system, nitrogen application rates were 305 kg N ha^−1^ yr^−1^ in China compared to 74 kg N ha^−1^ yr^−1^ worldwide^11^. However, the substantial increases in N input do not result in a corresponding increase in crop yields, and it costs unnecessary labor^1,9,12^. Instead, NUE declines greatly and large amounts of N are lost from the crop system^13–15^. Fortunately, in 2015, the Chinese Ministry of Agriculture issued Zero Increase Action Plan to curb the increase in fertilizer usage to zero by 2020^16^, which aims to increase crop yields without increasing fertilizer use, in an effort to reduce negative human impacts and environmental costs^15,16^. This plan was strongly supported by large-scale experiments of the three main staple crops (rice, wheat and maize) in China, which demonstrated that it is possible to reduce national fertilizer use while at the same time increase crop yields^9^. To achieve this goal, it is imperative to improve NUE and reduce N losses, without further increase the use of fertilizer^16^.

Among the fertilizers, N is the most important nutrient for maize (*Zea mays L*.) and can improve grain yield and quality of summer maize^5^. In China, a typical recommended N application rate may exceed 260 kg N ha^−1^ for summer maize^5,17^, whereas the region-wide experiments in the North China Plain have demonstrated that N rates could be reduced to 157 kg N ha^−1^ without a yield decrease^18^. In Anhui province, one of the provinces in the Huang-Huai-Hai Plain, maize is grown on about 110 million ha. The average N application rate of summer maize is more than 300 kg N ha^−1^, whereas the yield was only 5200 kg ha^−1^, lower than nearby provinces Shandong and Hebei^19^. Recently, Wang *et al*.^20^ reported that there was no significant increase in yield when N was applied at up to 300 kg N ha^−1^. The optimum N dose was 169 kg N ha^−1^ in Jianghuai area of Anhui province. Over-application of N fertilizer does not significantly increase maize yields but does result in accumulation of nitrate N (NO_3_-N) in the soil, and it causes large amounts of N to be lost to the environment^9,21^. Maize is often associated with large surpluses of soil mineral N that may reach 200 kg N ha^−1^ after harvest^22,23^. Therefore, it is essential to develop appropriate N management methods to reduce N losses and get a substantial and consistent yield increase of maize in this region. Such methods should not only optimize N inputs so that crops can achieve maximum yields and high NUE, but also minimize N losses to the environment.

Extensive studies have been performed to reduce N rate and enhance NUE, such as increasing the number of applications (split-surface broadcasting, SSB) according to the plant’s N needs^3,24,25^, using urease inhibitors and controlled-release fertilizer^26,27^, and planting more efficient maize varieties. Each method has a cost: urease inhibitors and controlled-release fertilizer are expensive; SSB applications are labor-intensive. The costs of farm labor in China are rising in general because the workforce has decreased as farmers have aged and people have moved to industrialized areas. Therefore, both farmers and the government are demanding simplified fertilization methods, such as one-time fertilization, provided the methods do not decrease crop yields^28^. Selecting the right fertilizer rate and placement is a key role into influencing plant growth and nutrient uptake, which are vital to reduce total N input and losses^29^. Identifying the optimum fertilization rate and placement has become extremely important for one-time fertilization programs. In several recent studies, one-time urea deep placement has been reported to increase crop yield and reduce N losses^30–32^. In our previous work, we also found that one-time nitrogen root-zone fertilization (RZF) significantly reduced N rates and N losses without reducing yields in rice paddy fields^28^ and the wheat–soil system^12^. RZF is a more exact deep placement of fertilization according to the specific crop^28,29,33,34^. For rice, fertilizer applied into 10 cm deep holes positioned 5 cm from the rice roots was the suitable RZF pattern, which increased rice yield while reduced fertilizer nitrogen loss^34^. For summer maize, we found that N applied all at one time as a basal fertilizer into a hole that was 5 cm from the seed and 12 cm deep was the effective RZF pattern for increasing nitrogen use efficiency and grain yield^33^. However, few studies have assessed the fate of isotopic urea (^15^N) or the N losses associated with RZF in summer maize drylands. The isotopic (^15^N) technique is widely used to evaluate the fate of fertilizer N and use efficiency in farmland ecosystems^2,3,12,22,25^. A better understanding of N fate in a summer maize cropping system would help improving N management for increasing maize yield while reducing N loss. Therefore, a 2-year consecutive field experiment was conducted to investigate the effect of RZF on maize yield, N uptake, NUE and the fate of N using the ^15^N tracer technique in Anhui province, one of the main maize planting regions of China.

## Results

### Maize yields

Across two years, the grain yield and biomass of maize was significantly affected by both application rate and method of N fertilizer (Table 1). Grain yield and biomass were significantly higher in all N application treatments (7.70–10.07 t ha^−1^ and 12.91–16.69 t ha^−1^, respectively) compared with the control treatment (5.47–5.68 t ha^−1^ and 8.92–9.69 t ha^−1^). Within the same application method (RZF or SSB), both grain yield and biomass increased with increased doses of N (from 0 to 225 kg ha^−1^). The grain yields for treatments N135 and N180 in RZF were significantly higher than SSB135 and SSB180 by 19.1% and 12.6%, respectively, in 2015, and by 15.5% and 8.1% in 2016. However, there was no significant difference in grain yield between SSB and RZF at the highest N dose (N225). Similarly, the biomass of RZF135 was 7.0% and 9.4% higher than that of SSB135, respectively, in 2015 and 2016, and 5.9% higher for RZF180 than SSB180 in 2016. Over all N doses, grain yield and biomass in RZF were 11.5% higher and 6.0% higher than in SSB, respectively.

**Table 1:** Effects of method of application of N and its dose on mean grain yield and mean biomass of summer maize in 2015 and 2016.

Treatments	N rate (kg ha^−1^)	2015		2016	
		Grain yield (t ha^−1^)	Biomass (t ha^−1^)	Grain yield (t ha^−1^)	Biomass (t ha^−1^)
CK	0	5.68 e	8.92 f	5.47 e	9.69 d
SSB	135	7.70 d	12.91 e	7.56 d	13.41 c
	180	8.62 c	14.03 cd	8.84 bc	15.30 b
	225	9.36 ab	15.05 b	9.40 ab	16.31 a
RZF	135	9.17 bc	13.88 d	8.73 c	14.67 b
	180	9.71 ab	14.80 bc	9.56 a	16.17 a
	225	10.07 a	15.91 a	9.97 a	16.69 a
Rate (R)		***	***	***	***
Method (D)		***	*	**	**
R × D		*	ns	ns	ns

Different small letters within the same column represent significant differences (*P* < 0.05). CK: N application 0 kg ha^−1^; SSB: two-split surface broadcasting; RZF: one-time root-zone fertilization. ns means not significant; *Significant at *P* < 0.05; **Significant at *P* < 0.01; ***Significant at *P* < 0.001.

### N uptake by maize

Both the N application rate and method significantly affected the N accumulation in both grain and the whole plant of maize (Table 2). The N accumulation in both grain and the whole plant was significantly higher in all N application treatments (81.4–116.4 and 144.3–191.4 kg ha^−1^, respectively) compared with the control (49.6–50.3 and 76.1–83.4 kg ha^−1^), and the N accumulation in both grain and the whole plant increased with increasing dosage of N. The N accumulation in the grain was significantly higher (16.1% at N135, 15.8% at N180, 15.0% at N225) in RZF than in SSB in 2015, however there was no difference for N180 or N225 in 2016. Similarly, the total N accumulation for in RZF135, RZF180 and RZF225 were significantly higher than in the same dosages of SSB (by 11.3%, 10.6% and 13.5%, respectively, in 2015, and by 7.8%, 7.3% and 9.3% in 2016).

**Table 2:** Effects of method of application of N and its dose on mean N uptake (kg ha^−1^) by maize in 2015 and 2016.

Treatments	N rate (kg ha^−1^)	2015		2016	
		Grain	Total	Grain	Total
CK		50.3 d	76.1 d	49.6 d	83.4 f
SSB	135	85.9 c	144.3 c	81.4 c	147.3 e
	180	97.7 b	164.6 b	95.2 b	167.6 cd
	225	101.2 b	168.6 b	99.0 ab	174.3 bc
RZF	135	99.7 b	160.6 b	95.2 b	158.8 d
	180	113.1 a	182.0 a	102.3 ab	179.7 ab
	225	116.4 a	191.4 a	108.3 a	190.5 a
Rate (R)		***	***	***	***
Method (D)		***	***	**	**
R × D		ns	ns	ns	ns

Different small letters within the same column represent significant differences (*P* < 0.05). CK: N application 0 kg ha^−1^; SSB: two-split surface broadcasting; RZF: one-time root-zone fertilization. ns means not significant; **Significant at *P* < 0.01; ***Significant at *P* < 0.001.

### Nitrogen use efficiency

All three NUE indexes [the N apparent recovery efficiency (NARE), N agronomic efficiency (NAE), and N partial factor productivity (NPFP)] were significantly affected by fertilization method over two consecutive years. NARE and NPFP were also significantly affected by N application rate in both years, whereas NAE was not affected by the N application rate in 2016 (Table 3). NARE in RZF was 51.2–62.8% and 47.6–55.9% in 2015 and 2016, respectively, which increased by 19.7–26.5% (in 2015) and 14.3–18.2% (in 2016) compared with SSB (Table 3). Application method had a similar effect on NAE, NPFP and NARE; all indexes were significantly higher in RZF than SSB in both seasons.

**Table 3:** Effects of method of application of N and its dose on three measures of N use efficiency (NUE) in 2015 and 2016.

Treatments	N rate (kg ha^−1^)	2015			2016		
		NARE (%)	NAE (kg kg^−1^)	NPFP (kg kg^−1^)	NARE (%)	NAE (kg kg^−1^)	NPFP (kg kg^−1^)
SSB	135	49.5 b	14.9 c	57.0 b	47.3 b	15.5 c	56.0 b
	180	49.2 b	16.3 c	47.7 c	46.8 b	18.7 b	50.5 c
	225	41.1 c	16.4 c	41.6 d	40.4 c	17.5 bc	41.8 d
RZF	135	62.6 a	25.8 a	67.9 a	55.9 a	24.1 a	64.6 a
	180	58.9 a	22.4 b	54.3 b	53.5 a	21.0 a	52.7 bc
	225	51.2 b	19.5 b	44.8 cd	47.6 b	19.6 b	44.3 d
Rate (R)		**	ns	***	**	ns	***
Method (D)		***	***	***	***	***	***
R × D		ns	**	*	ns	**	ns

Different small letters within the same column represent significant differences (*P* < 0.05). CK: N application 0 kg ha^−1^; SSB: two-split surface broadcasting; RZF: one-time root-zone fertilization. ns means not significant; *Significant at *P* < 0.05; **Significant at *P* < 0.01; ***Significant at *P* < 0.001.

### Plant nitrogen derived from fertilizer and soil

The N derived from fertilizer (Ndff) in grain and whole plant were significantly affected by the method and rate of N fertilizer. As showed in Table 4, the Ndff in both grain and the whole plant increased with increasing the application dose of N. RZF significantly increased the Ndff in grain and the whole plant by 12.6–28.5% and 22.0–30.1% compared with SSB, respectively, and increased the Ndff (%) by 13.1–19.6%. However, the percentage N derived from soil (Ndfs) for RZF was significantly lower than that of SSB treatments. In general, RZF significantly increased Ndff (%) but decreased Ndfs (%), compared with SSB, and the proportion of fertilizer uptake at the highest doses (N225) was higher than at the lowest N doses (N135).

**Table 4:** Maize plant N derived from ^15^N-labeled urea (Ndff) and from soils (Ndfs) in 2016.

Treatments	N rate (kg ha^−1^)	Ndff (kg ha^−1^)		Ndfs (kg ha^−1^)		Ndff (%)	Ndfs (%)
		Grain	Total	Grain	Total		
SSB	135	15.8 e	27.9 e	63.1 b	124.1 c	18.4 e	81.6 a
	180	25.5 c	42.7 c	73.4 a	135.9 abc	23.9 cd	76.1 bc
	225	25.9 c	46.7 c	73.3 a	138.6 ab	25.2 bc	74.8 cd
RZF	135	20.3 d	36.3 d	75.0 a	128.6 bc	22.0 d	78.0 b
	180	28.7 b	52.1 b	75.2 a	139.2 ab	27.5 ab	72.7 de
	225	31.8 a	57.8 a	77.7 a	145.2 a	28.5 a	71.5 e
Rate (R)		***	***	ns	*	***	***
Method (D)		***	***	*	ns	***	***
R × D		ns	ns	ns	ns	ns	ns

Different small letters within the same column represent significant differences (*P* < 0.05). CK: N application 0 kg ha^−1^; SSB: two-split surface broadcasting; RZF: one-time root-zone fertilization. ns means not significant; *Significant at *P* < 0.05; ***Significant at *P* < 0.001.

### Distribution of residual urea-^15^N in soil

**Figure 1:**
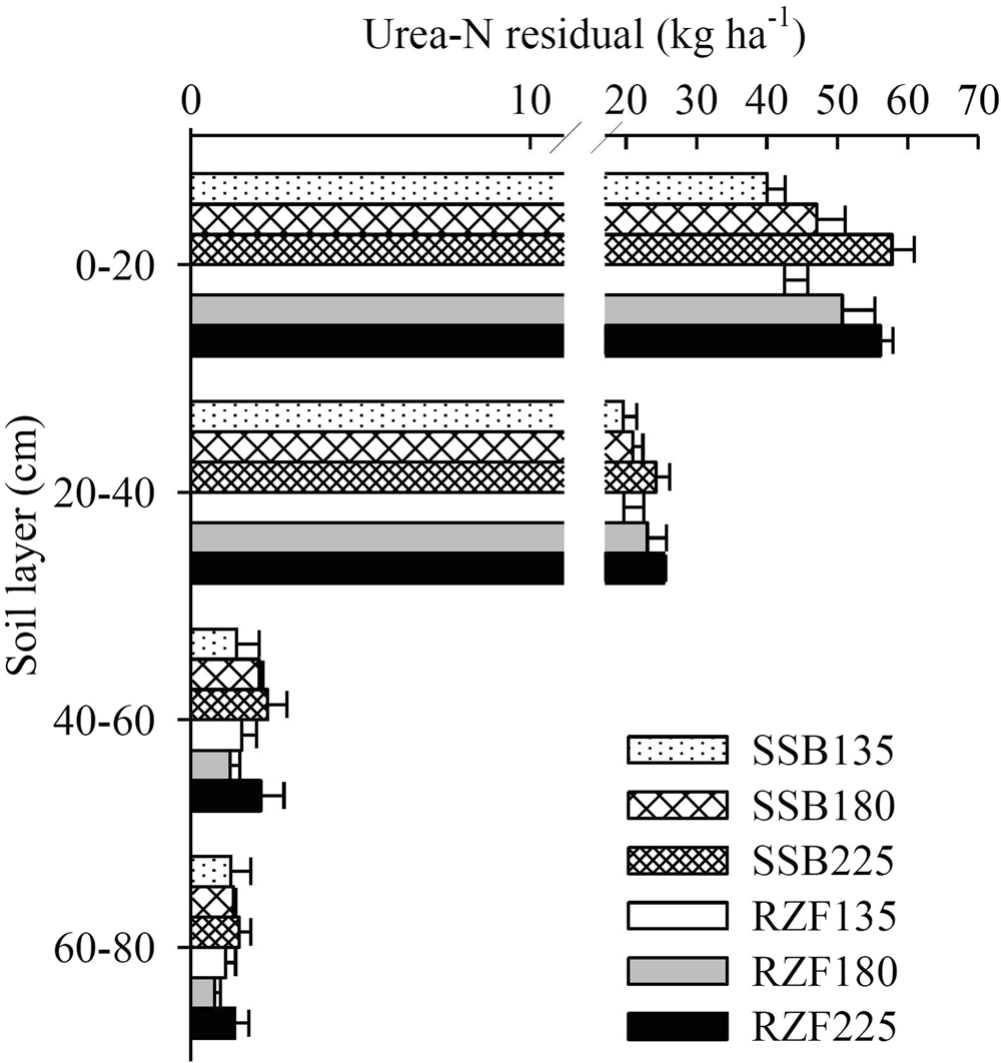
The distribution of residual ^15^N after harvest as a factor of application method and rate of N in 2016. SSB135, SSB180 and SSB225: two-split surface broadcasting with 135, 180 and 225 kg N ha^−1^, respectively; RZF135, RZF180 and RZF225: one-time root-zone fertilization with 135, 180 and 225 kg N ha^−1^, respectively.

At harvest, the total residual ^15^N-labeled urea in the 0–80 cm soil profile layer range was 61.3–64.0 (N135), 70.5–75.1 (N180), and 83.7–84.2 kg ha^−1^ (N225), and accounted for 45.4–47.4%, 39.2–41.7%, and 37.2–37.4% of ^15^N, respectively (Fig. 1). The N application method and N dose did not significantly affect total residual ^15^N in the 0–80 cm soil profile layer. In general, the total residual ^15^N accumulation in the 0–80 cm soil profile layer decreased with increasing dosage of N. A major portion of ^15^N-labeled fertilizer was in the top layer of soil (0–20 m), accounting for 64.4–67.4% of total residual N in soil (Fig. 1).

### Fate of urea-^15^N in maize–soil system

The N application method significantly affected the N recovery in maize and potential losses, but did not affect the N residual in soil (Table 5). Under the same N dosage, the N recovery in RZF (36.3–57.8 kg ha^−1^, accounting for 25.7–28.9%) was significantly higher than it was in SSB (27.9–46.7 kg ha^−1^, accounting for 20.7–23.7%). The N recovery in RZF was significantly higher than in SSB by 30.0% (N135), 21.9% (N180), and 23.6% (N225). In contrast, the potential losses of N were significant lower in RZF (25.7–37.1%) than in SSB (33.9–41.8%). The potential losses of N for RZF were reduced by 24.2% (N135), 20.8% (N180), and 11.2% (N225) compared with the SSB treatment. Overall, RZF had greater N recovery, while reducing the potential losses of N; and the potential losses of N was increased with increasing application dose of N in both RZF and SSB treatments.

**Table 5:** The fate of ^15^N-labeled urea in the 2016 maize growing seasons.

Treatments	N rate (kg ha^−1^)	^15^N in maize (kg ha^−1^)	^15^N in soil (kg ha^−1^)	Recovery in maize (%)	Residual in soil (%)	Potential losses (%)
SSB	135	27.9 e	61.3 d	20.7 d	45.4 ab	33.9 bc
	180	42.7 c	70.5 bc	23.7 c	39.2 c	37.1 ab
	225	46.7 c	84.2 a	20.8 d	37.4 c	41.8 a
RZF	135	36.3 d	64.0 cd	26.9 ab	47.4 a	25.7 d
	180	52.1 b	75.1 b	28.9 a	41.7 bc	29.4 cd
	225	57.8 a	83.7 a	25.7 bc	37.2 c	37.1 ab
Rate (R)		***	ns	***	**	**
Method (D)		***	***	**	ns	***
R × D		ns	ns	ns	ns	ns

Different small letters within the same column represent significant differences (*P* < 0.05). CK: N application 0 kg ha^−1^; SSB: two-split surface broadcasting; RZF: one-time root-zone fertilization. ns means not significant; **Significant at *P* < 0.01; ***Significant at *P* < 0.001.

## Discussion

RZF increased maize grain yield by an average of 11.5% compared with SSB over two consecutive years. In our previous studies, rice yield of one-time RZF of N was 19.5% greater than the yield for 3-split surface broadcasting^34^. Significant increase in crop yield for improved fertilization method have been reported in both rice and wheat compared with surface broadcasting^15,22^. Deep N application (10 cm) could increase wheat yields by 30.3% on average compared with surface broadcasting^22^. Yao *et al*.^15^ found that one-time deep placement of urea increased rice yields by 10% compared with surface broadcasting. Interestingly, grain yield of RZF with low N doses (135 kg N ha^−1^) was equal to that of SSB with middle N doses (180 kg N ha^−1^), and grain yield of RZF180 was also equal to that of SSB225 in 2016, and even 11% higher than that of SSB225 in 2015. That is, when the rate of N application was reduced 20–25% in the root-zone fertilization, RZF135 and RZF180 still produced almost the same yield of maize grain as SSB180 and SSB225, respectively, in which urea was split-fertilized. Under the low and middle N doses (135 and 180 kg N ha^−1^), RZF significantly increased grain yield by 11.8–15.9% compared with SSB, whereas there was no significant difference between RZF and SSB for the high N doses (225 kg N ha^−1^) application.

**Figure 2:**
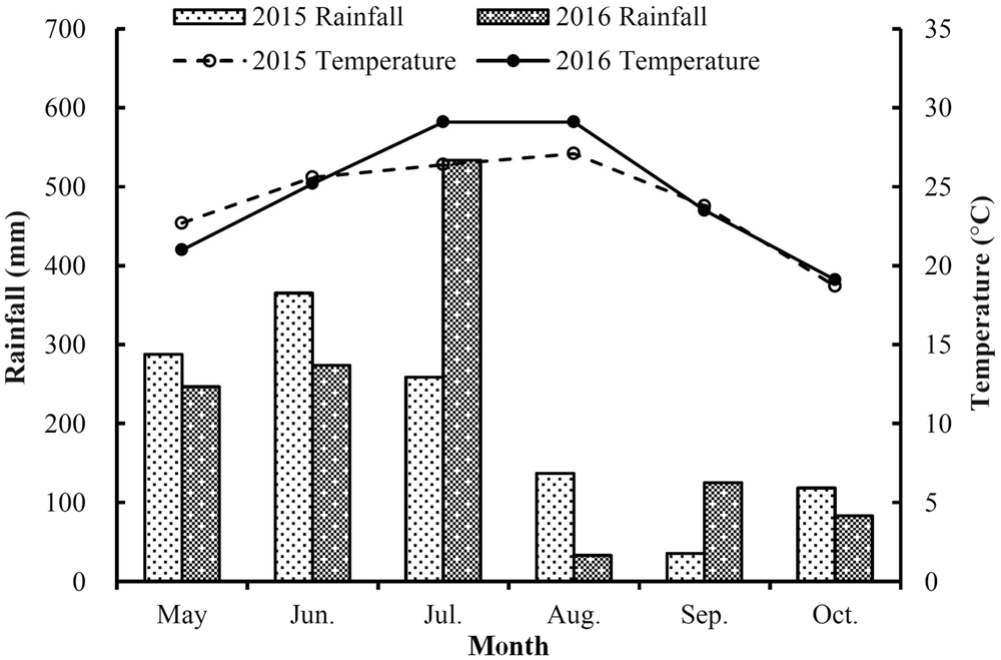
Monthly mean rainfall and temperature during the maize growing season in 2015 and 2016.

Maize yields varied between 2015 and 2016, especially among the RZF treatments, which may have been driven by environmental conditions, particularly high temperature and heavy rainfall. In the 2016 maize season, the temperature (29.1 °C on average) in July was higher than that in 2015 (26.4 °C). The rainfall in July 2016 (533.8 mm) was extremely higher than that in July 2015 (259.1 mm). More importantly, the rainfall in the first 8 days of July was 487.6 mm (91% of this month), particular in July 3, the rainfall reached up to 253.2 mm (Fig. 2). Heavy rainfall would lead to serious N leaching to the deeper soil layers which cannot be absorbed by maize roots. And therefore, the grain yield of RZF was limited by the reduction in nitrogen absorption in 2016. However, half of N fertilizers for SSB were applied on July 8, 2016 (45 days after sowing). Therefore, the N absorption for plant in SSB was less inhibited by rainfall than that in RZF, and the grain yield for SSB kept in a relatively stable level during 2015 and 2016. This may explain why the available N of RZF treatments in 2016 was lower than it was in 2015. Yields in the RZF treatment were slightly higher in 2015 compared with 2016, whereas yields in the SSB treatments were not significantly different between 2015 and 2016 (Table 1). In general, one-time RZF of urea achieved higher grain yields and biomass than 2-split surface broadcasting for the summer maize system used in this study, especially in the low and middle N application rates. Our study suggests that one-time RZF can be an effective way to reduce the amount of N fertilizer, which deserves more attention and further research in the future.

The N accumulation in plant (grain and straw) was 144.3–191.4 kg ha^–1^ in the N application treatments, which was similar to the results in the adjacent area of this study^35,36^. Zheng *et al*.^36^ reported that N accumulation for the whole plant was 150.1–176.5 kg ha^−1^ in the N application treatments in region along Huai River of Anhui province. However, RZF increased the total uptake of N by 7.2–13.5% compared with SSB treatments, indicating that RZF of urea could greatly promote fertilizer N uptake. This is consistent with previous studies by Liu *et al*.^28^ and Yao *et al*.^15^, who found that one-time RZF or urea deep placement significantly increased N uptake in rice compared with 3-split surface broadcasting treatments. Based on two-year study, the NH_4_^+^-N content was higher in the root zone than that of surface broadcasting during the early growth stage, which supplied continuous high levels of N for plants, and thus promoted root growth and nutrient uptake under RZF and deep placement of urea^15,28^. In contrast, when urea was broadcast onto the soil surface, it was rapidly hydrolyzed to root growth-stimulating NH_4_^+^ and lost through NH_3_ volatilization and runoff^15,32^. Rees *et al*.^22^ also found that N uptake by the grain and the whole maize plant in point deep placement increased by 32.5% and 7.9%, respectively, compared with surface broadcasting. Urea deep placement could minimize NH_3_ volatilization, denitrification and runoff compared with surface broadcasting^15,37,38^, thereby reducing N losses and increasing N uptake by plants. However, further studies on the root spatial distribution and N uptake, and their interaction on increasing maize yield under RZF are needed.

Furthermore, using the ^15^N tracer technique, we monitored the uptake of N by plants to determine whether N was coming from the soil or from fertilizer. We found that the Ndff (%) in whole plant tissues averaged 18.4–28.5% compared with 71.5–81.6% of plant N derived from soil (Table 4). This is consistent with previous studies by Wang *et al*.^3^ and Rimski-Korsakov *et al*.^39^, who reported that 67.6–73.2% and 54–78%, respectively, of plant N coming from the soil. Chen *et al*.^40^ also found that N from soil organic matter contributed approximately 83% of the total N in the rice–wheat cropping system, and the proportion was about 88% when crop residues were applied. These results suggest that the mineralization of soil organic matter is an important source of N for crops. However, RZF significantly increased the Ndff (%) by 13.1–19.6% compared with SSB, while the Ndfs (kg ha^−1^) in RZF was not significantly different than that of SSB (*p* > 0.05) under the same N application rate (Table 4). That is, one-time urea RZF significantly reduced nitrogen losses without depleting soil nitrogen. This finding was in accordance with Rees *et al*.^22^, who found that point placement significantly increased the Ndff (%) of summer maize by 27.8–33.3% compared with surface application. Recently, Yao *et al*.^15^ also reported that one-time urea deep placement significantly increased the Ndff (%) of rice by 62% compared with surface broadcasting. Urea deep placement could maintain a significant higher NH_4_^+^-N content than surface broadcasting in the root zone^15,28,41^. When roots encounter a nutrient-rich zone or patch, they can enhance its proliferation and the capture capabilities of fertilizer nutrients^38,41^. In contrast, when urea was broadcast onto surface, the nitrogen nutrient was easily hydrolyzed to NH_4_^+^ and lost through NH_3_ volatilization and runoff, because the fertilizer nutrients stayed away from the roots^15,41^. Thereby, the N uptake derived from fertilizer was significant higher in RZF than that in SSB. Overall, these results indicate that RZF of urea could greatly enhance the plant N uptake from fertilizer and N use efficiency.

RZF significantly increased NARE and N recovery by 14.3–37.8% and 21.9–30.0%, respectively, compared with SSB (Tables 3 and 5). This is consistent with the findings of Rees *et al*.^22^, who reported that the maize recovery of fertilizer N following point placement was 38.9% higher than for surface application. Similarly, previous studies reported that deep placement of N fertilizer could increase N recovery efficiency (NRE) of rice by 26–93%, and significantly increase ^15^N recovery, compared with surface broadcasting^15,28,41^. Our previous study carried out in the typical lime concretion black soil (heavy texture) also showed consistent results as the present study (silty clay)^42^. It has been reported that deep placement of urea increases growth of lateral roots at deep soil layers and also increases taproot diameter and length^43^. Deep placement of urea has also been shown to result in persistently high levels of nutrients in plant-available form close to roots^32^, thereby increasing uptake by plants. Moreover, deep-placed urea decreases NH_3_ volatilization, denitrification and runoff compared surface broadcasting^15,37,38^, and thus reducing N losses and increasing N uptake by plants. Moreover, deep placement increased the NAE and NPFP of maize under the same rate of N application (Table 3), which was in agreement with the results described by Liu *et al*.^41^, who reported that deep placement of N significantly increased NAE and NPFP by 31–51% and 10–16%, respectively, in paddy fields compared with surface broadcasting. Recently, Pan *et al*.^44^ also found that mechanical deep placement of N fertilizer significantly enhanced NAE by 19.5–50.4% in a rice cropping system of South China, compared with surface broadcasting. Therefore, RZF is an effective way to improve NUE for maize systems.

The potential N losses were significantly reduced in the RZF treatments. Lower potential N losses for deep placement also found by Cai *et al*.^38^, who reported that deep placement greatly reduced the total N loss from 42–67% to 10–27% compared with surface broadcasting. The N loss fertilizer is closely related to the application method and N rate^3,6,15^. Previous studies have reported that the N loss was significant lower with one-time deep placement than split-surface broadcasting for rice^15,28,40^. However, Chen *et al*.^45^ reported that compared to 3-split application, unaccounted-N loss in winter wheat for one-time band deep placement was increased by 21.7%. Therefore, optimal one-time deep application may be different for different crop to decrease N loss. Lower fertilizer N loss for RZF treatments may be due to a slow-release fertilizer, which better matches the N demand of maize plants and remarkably shrinks NH_3_ volatilization^15,28^. In contrast, there was no significant difference of the total residual ^15^N between RZF and SSB at the same application rates of N (Table 5). At harvest, the total residual ^15^N-labeled fertilizer in the 0–80 cm soil layer was 37.2–47.4% of ^15^N fertilizer, with 64.4–67.4% of that retained in the 0–20 cm layers (Fig. 1 and Table 5). These numbers are similar to those of Wang *et al*.^3^ and Yang *et al*.^46^, who found that about half of total residual ^15^N-labeled fertilizer retained in the 0–20 cm soil layer at maize harvest. For wheat, most of the residual N (76.8–87.0%) was retained in the 0–20 cm soil layer in southeastern China^45^. These results indicate that residual N does not move much, and the N leaching risk was low. In general, nitrogen can be lost mainly through ammonia volatilization, runoff, leaching and denitrification^15,38^. However, we just only investigated the total potential losses of the SSB and one-time RZF of urea in the present study. Therefore, more studies should be carried out to directly measure the N losses, such as ammonia volatilization, runoff, leaching and denitrification in future. Nevertheless, RZF is a method for improving the efficiency of fertilizer application and achieving low levels of leaching, while there is room to further develop and apply root-zone fertilization machinery.

## Conclusions

RZF increased maize grain yields by an average of 11.5% compared with SSB, and RZF achieved high yield as SSB with a 20–25% reduction in the N dose under 135–180 kg N ha^−1^. The plant N derived from fertilizer averaged 18.4–28.5% compared with 71.5–81.6% derived from soil. RZF significantly increased the Ndff (%) by 13.1–19.6% compared with SSB. The total residual ^15^N in the 0–80 cm soil depth was 37.2–47.4% after maize harvest; most residual ^15^N (64.4–67.4%) was retained in the top 20 cm. RZF significantly increased the NARE and N recovery in maize by 14.3–37.8% and 21.9–30.0%, respectively, compared with SSB. Moreover, RZF decreased the potential losses of N by 11.2–24.2% compared with SSB. Overall, our study suggests that one-time RZF can improve crop yields and minimize nitrogen loss in summer maize in China.

## Materials and Methods

### Experimental site

A two-year experiment was conducted in Dongzhi county (30°17′N, 117°4′E), in Anhui province, southeastern China. The region has a subtropical, humid monsoon climate. The annual mean air temperature and the average annual precipitation from 1951 to 2008 are 16.9 °C and 1554 mm, respectively. The pattern of monthly mean air temperature and precipitation during the maize growing season from 2015 to 2016 is given in Fig. 2. The soil of the experimental site is classified as a red-yellow soil. The texture of the soil is silty clay (international system) and the dominant fraction is silt. The physicochemical properties of the topsoil (0–20 cm) are pH of 5.7 (H_2_O), 17.0 g kg^−1^ organic matter, 0.76 g kg^−1^ total N, 76.6 mg kg^−1^ available N, 28.1 mg kg^−1^ available P, and 112.5 available K, 1.27 g cm^−3^ soil bulk density.

### Experiment design and agricultural management practices

The experiment was conducted in two consecutive seasons, in 2015 and 2016. The field plot experiment included seven treatments, each with three replicates arranged in the field in a completely randomized block design. The control (CK) had no added N; the 2-split surface broadcasting (SSB) treatments had N doses of 135, 180 or 225 kg ha^−1^ (SSB135, SSB180 and SSB225, respectively); and one-time RZF treatments also had N doses of 135, 180 or 225 kg ha^−1^ (RZF135, RZF180 and RZF225, respectively).

The size of each plot was 6.7 m^2^ (2.8 m × 2.4 m) in 2015 (40 plants for each plot) and 16.8 m^2^ (5.6 m × 3.0 m) in 2016 (100 plants for each plot). In the 2016 season, a small sub-plot for ^15^N analyses was set up within the plots. The sub-plot was 0.5 m^2^ (0.84 m × 0.6 m), and included three plants. Each plant fertilized with ^15^N was bordered by 50 cm-high polyvinyl chloride (PVC) frame (28 cm-wide and 60 cm-long) with 45 cm inserted into the soil and 5 cm exposed on the surface to prevent runoff and lateral contamination. We used the maize cultivar ‘Longping 206’, a local prevailing cultivar. Seeds were sown at a spacing of 60 cm × 28 cm (60,000 plants ha^−1^) in all treatments. The ^15^N-labeled urea (46% N content, and 10.15% of ^15^N abundance ratio) was provided by the Shanghai Research Institute of Chemical Industry. The non-labeled N fertilizer was urea (46% N). The P fertilizer (135 kg P_2_O_5_ ha^−1^, superphosphate) and K fertilizer (180 kg K_2_O ha^−1^, potassium chloride) were applied to all treatments at the time of sowing. In the RZF treatments, the urea was point deep-placed all at one time as a basal fertilizer (4.89, 6.52 and 8.15 g urea plant^−1^ for RZF135, RZF180 and RZF225, respectively). Both the ^15^N-labeled and non-labeled urea for RZF were manually placed into a 12-cm deep hole positioned 5 cm from each maize seed using a steel-pipe tool (a structure similar to an injection syringe). In the SSB treatments, 50% non-labeled or ^15^N-labeled urea was broadcast by hand at sowing, and 50% at tasseling stage. P and K fertilizers were broadcast as basal fertilizers in all treatments.

Plants were watered in accordance with typical irrigation practices, and irrigation practices were the same for all treatments. Pesticide and herbicide applications were the same for all treatments. Maize was sown by hand on May 29, 2015 and May 24, 2016, and harvested on Sept. 22, 2015 and Sept. 19, 2016.

### Plant and soil sampling and analysis

Plants in each plot were harvested close to the ground, and then were separated into straw and grain. Two plants from each plot that had been labeled ^15^N urea were harvested and separated into straw, grain, and roots; the roots were collected from the 0–60 cm soil layer and washed. The dry weight was determined after drying at 70 °C to constant weight. An aliquot of each dry sample was ground to powder and passed through a 0.15-mm sieve in preparation for total N content and isotopic ^15^N analyses. Soils were sampled to a depth of 80 cm; each soil sample had a radius of 20 cm and was divided into 20 cm sections. Total N of the grain, straw and roots were analyzed using the Kjeldahl method. The ^15^N abundance was measured using an elemental analyzer (Costech ECS4010, Costech Analytical Technologies Inc., Valencia, USA) coupled to an isotope ratio mass spectrometer (Delta V Advantage, Thermo Fisher Scientific Inc., USA). Soil bulk density was determined after harvesting with the cutting ring method.

### Calculation methods

All ^15^N was expressed as atom percent excess and corrected for background abundance (at 0.366%). The N derived from fertilizer (Ndff) and N derived from soil (Ndfs) were calculated according to the following equation^3,15^:

1 $${\rm{Ndff}}\,( \% )=\frac{{\rm{B}}-{\rm{A}}}{{\rm{C}}-{\rm{A}}}\times 100$$

where A is the ^15^N natural abundance; B is the atom percent excess of ^15^N in the plant or soil; and C is the atom percent excess of ^15^N in the fertilizer N.

2 $${\rm{Plant}}\,{\rm{Ndff}}\,({\rm{kg}}\,{{\rm{ha}}}^{-1})={\rm{Ndff}}\,( \% )\times {\rm{plant}}\,{\rm{total}}\,{\rm{N}}\,{\rm{content}}\times {\rm{plant}}\,{\rm{dry}}\,{\rm{matter}}\,{\rm{biomass}}$$

3 $${\rm{Soil}}\,{\rm{Ndff}}\,({\rm{kg}}\,{{\rm{ha}}}^{-1})={\rm{Ndff}}\,( \% )\times {\rm{soil}}\,{\rm{total}}\,{\rm{N}}\,{\rm{content}}\times {\rm{soil}}\,{\rm{bulk}}\,{\rm{density}}\times {\rm{soil}}\,{\rm{thickness}}$$

4 $${\rm{Ndfs}}\,({\rm{kg}}\,{{\rm{ha}}}^{-1})={\rm{plant}}\,{\rm{total}}\,{\rm{N}}\,{\rm{content}}\times {\rm{plant}}\,{\rm{dry}}\,{\rm{matter}}\,{\rm{biomass}}\,-\,{\rm{Ndff}}$$

5 $${\rm{N}}\,{\rm{recovery}}\,{\rm{in}}\,{\rm{plant}}\,( \% )=(2)/{\rm{N}}\,{\rm{application}}\,{\rm{rate}}\times 100$$

6 $${\rm{N}}\,{\rm{residual}}\,{\rm{in}}\,{\rm{soil}}\,( \% )=(3)/{\rm{N}}\,{\rm{application}}\,{\rm{rate}}\times 100$$

7 $${\rm{Potential}}\,{\rm{losses}}\,( \% )=100\,-\,(5)\,-\,(6)$$

The N fertilizer accumulation and recovery by maize were calculated according to the methods of Wang *et al*.^3^. Nitrogen apparent recovery efficiency (NARE), nitrogen agronomic efficiency (NAE), and nitrogen partial factor productivity (NPFP) were calculated according to the following method^16,41,44^.

8 $${\rm{NARE}}\,( \% )=\frac{{{\rm{U}}}_{{\rm{N}}}\,-\,{{\rm{U}}}_{0}}{{{\rm{F}}}_{{\rm{N}}}}\times 100 \% $$

9 $${\rm{NAE}}\,({\rm{kg}}\,{{\rm{kg}}}^{-1})=\frac{{{\rm{Y}}}_{{\rm{N}}}\,-\,{{\rm{Y}}}_{0}}{{{\rm{F}}}_{{\rm{N}}}}$$

10 $${\rm{NPFP}}\,({\rm{kg}}\,{{\rm{kg}}}^{-1})=\frac{{{\rm{Y}}}_{{\rm{N}}}}{{{\rm{F}}}_{{\rm{N}}}}$$

where U_0_ and U_N_ represent the total N uptake (kg ha^−1^) by the grain and straw in the N_0_ plot and other N-fertilized plots, respectively; Y_0_ and Y_N_ are the grain yield (kg ha^−1^) in the N_0_ plot and other N-fertilized plots, respectively; and F_N_ is the rate of applied fertilizer N (kg ha^−1^).

### Statistical analysis

Statistical analysis was performed using SPSS 19.0 (SPSS Inc., Chicago, IL, USA). Two-way analysis of variance (ANOVA) was used to assess the effects of N fertilizer placement and N rate on maize yield, N uptake, and the fate of urea ^15^N. The treatments were compared by the method of least significance difference at p < 0.05.
